# Nano-Enhanced Optical Delivery of Multi-Characteristic Opsin Gene for Spinal Optogenetic Modulation of Pain

**DOI:** 10.3390/bioengineering13040479

**Published:** 2026-04-20

**Authors:** Darryl Narcisse, Robert Benkowski, Matthew Dwyer, Samarendra Mohanty

**Affiliations:** 1 Opsin Biotherapeutics, Inc., 1312 Brown Trail, Bedford, TX 76022, USA; 2 DesignPlex Biomedical, LLC, 3425 Clayton Rd. E, Fort Worth, TX 76116, USA; 3 Nanoscope Technologies, LLC, 1624 New York Ave., Arlington, TX 76010, USA; m.blake.dwyer@tcu.edu

**Keywords:** optogenetics, pain, spine, neuromodulation, multi-characteristic opsin, nano-enhanced optical delivery

## Abstract

Optogenetic modulation employs light-sensitive proteins known as opsins to regulate cellular activity. A unique therapeutic application of this technique involves modulating pain perception by selectively targeting neural pathways within the spinal cord. Multi-Characteristic Opsin (MCO) represents an innovative optogenetic actuator capable of activation across a broad spectrum of light wavelengths, exhibiting a slow depolarizing phase that resembles natural photoreceptors. This study examines the current advancements in spinal optogenetic modulation utilizing MCO for pain management. Due to its high sensitivity, MCO facilitates minimally invasive, remotely controlled optogenetic modulation of spinal neurons. This approach enables the regulation of extensive spatial regions, provided the MCO channel receives sufficient light intensity to surpass the activation threshold. Nano-enhanced optical delivery (NOD) successfully transfected spinal neurons with the GAD67-MCO2-mCherry construct, as confirmed by membrane-localized mCherry fluorescence with DAPI-labeled nuclei. Using this platform, 5 Hz spinal optogenetic stimulation produced a significant reduction in formalin-evoked pain behaviors, demonstrating frequency-specific modulation of spinal pain circuits. Neither 2 Hz nor 10 Hz stimulation yielded comparable analgesic effects, underscoring the importance of precise stimulation parameters. The therapeutic impact also depended on transfection efficiency: reducing the fGNR–plasmid concentration diminished MCO expression and weakened the analgesic response. Together, these results show that effective spinal optogenetic pain modulation requires both optimal stimulation frequency and robust gene delivery.

## 1. Introduction

Chronic pain represents a pervasive medical condition that negatively impacts millions of individuals in the United States and worldwide [1,2,3], presenting considerable challenges in effective treatment. It remains a primary cause of long-term disability, leading to increased healthcare expenditures, reduced occupational productivity, and substantial declines in quality of life [4,5,6,7,8]. Furthermore, chronic pain is frequently associated with comorbid mental health conditions, notably anxiety and depression, and disproportionately affects vulnerable populations, such as individuals living in poverty or rural settings [9,10].

Existing therapeutic strategies, including opioid analgesics and electrical spinal cord stimulation, are often limited by significant adverse effects and suboptimal efficacy [11,12]. Many current standard pain interventions lack precision, as they are unable to selectively target specific neuronal subpopulations. When standard pharmacological therapies prove ineffective, approximately 21% of patients with neuropathic pain develop a marked dependence on opioid medications [13], consequently increasing the risk of addiction and overdose-related mortality.

To address these limitations, optogenetic methodologies employ promoter-specific expression of opsins within targeted neuronal populations, such as GABAergic neurons. Research underscores the critical function of GABAergic neurons within the dorsal horn of the spinal cord in integrating pain information [14].

In contrast to traditional spinal cord stimulation, optogenetics allows highly specific [15] neuromodulation. Optogenetics utilizes a class of protein channels known as opsins, which can be paired with expression regulation elements like promoters to limit expression to specific cellular populations. In doing so, these cells become sensitive to the wavelengths and intensity of light to which the opsin is responsive. This may lead to several secondary outcomes, including either an increase or a decrease in the neuron’s membrane potential, thereby facilitating or suppressing its capacity to generate action potentials. When expressed within key communicating or regulatory cells of the pain pathway, this offers the potential for optogenetic control of the pain experience, leading to sustainable analgesia. As such, optogenetics constitutes a promising advancement in the therapeutic management of chronic pain.

Recent advances in optogenetic technology have facilitated the selective stimulation of defined neuronal populations with millisecond accuracy, thereby allowing for the intricate dissection of neural pathways involved in nociception and pain processing [16]. Classical opsins, however, typically require stimulation with high-intensity blue light [17]. This constraint poses a significant risk for tissue phototoxicity and may induce inflammatory responses [18,19], thereby limiting the clinical applicability of traditional optogenetic approaches. To address these challenges, the Multi-Characteristic Opsin (MCO), a novel hybrid opsin engineered for activation by low-intensity red light, was developed [20]. The use of red-sensitive opsins such as MCO minimizes the risk of collateral tissue damage while maintaining robust efficacy in neural modulation. We proposed that nano-enhanced MCO delivery to spinal inhibitory interneurons could allow low-intensity red-light optogenetic stimulation to suppress pain signaling, with analgesic effectiveness depending on stimulation frequency.

In the present study, MCO was delivered to the spinal cord of murine models using a non-viral laser-based transfection method [21]. Optogenetic stimulation was then administered using a red light-emitting diode (LED) to precisely control the temporal dynamics of neuronal activation. The use of this technique provides a localized expression of opsin-encoding DNA based on the area of effect of the irradiation. Any DNA that is not taken up into target cells should easily be cleared in any organism with properly functioning microglia and DNAse [22,23]. Via this technique, a single injection can induce light-responsiveness in the key area of the CNS with no requirement for an invasive implant and highly efficient delivery. Pain-related behaviors were systematically evaluated using the formalin test to model nociceptive and inflammatory pain. This represents a novel approach of combining light-based delivery and light-based neuromodulation in the literature.

## 2. Materials and Methods

### 2.1. Ethics Statement

This study was conducted in strict accordance with the recommendations in the Guide for the Care and Use of Laboratory Animals of the National Institutes of Health. The Institutional Animal Care and Use Committee (IACUC) of Nanoscope Technologies, LLC approved the protocol (Pain_NS_2016). Essential methods (e.g., isoflurane and rest/standard analgesia when outside experimental norms) were used to minimize suffering, except when pain was necessary for the experiment.

### 2.2. Experimental Animal Selection and Care

All experimental mice were C57BL/6J (B6) inbred mice selected from related litters kept at the Nanoscope colony. According to our standard, at least 4 mice would be sufficient to show trends for each treatment type in this experiment. Eight adult mice were randomly selected among cage litters that were at least 8 weeks old to allow for potential losses over the course of the experiment. The control mice and experimental mice were all kept under the same conditions during experimentation. Rodents were kept on a standard 12:12 day-night cycle. All experiments were conducted per the standards as put forth by the Nanoscope IACUC. No initial selection criteria were established, and exclusion occurred in the case of eventualities (e.g., injury or death), which prevented experimentation or would have affected results. As per the Nanoscope IACUC, outside of experimental requirements, standard treatment, including rescue analgesia, was available for care of any observed injury, illness, or pain outside of testing.

### 2.3. Intrathecal Injection Procedure

This procedure was adapted from the siRNA delivery method for dorsal root ganglion (DRG) expression described by Njoo et al. (2014) [24]. Rodents were anesthetized with 2–3% isoflurane until the righting reflex was absent. Animals were then placed in a nose cone to maintain anesthesia, with isoflurane concentration reduced to 1.5–2%. Tail and paw pinch reflexes were periodically evaluated to ensure a suitable depth of anesthesia. Fur in the posterior region (~2 cm^2^) near the base of the tail was removed using a chemical depilatory agent, and the area was disinfected with 70% ethanol. The L6 spinous process was identified as an anatomical landmark, and a needle was inserted with the bevel facing upward into the intervertebral space between the L5 and L6 vertebrae. Proper needle placement in the intrathecal space was confirmed by observing a characteristic tail flick response. The syringe was secured using a magnetically secured arm, and the mixture of fGNRs and MCO-encoding DNA was administered slowly into the intrathecal space.

### 2.4. Nano-Enhanced Optogenetic Delivery (NOD)

**Figure 1:**
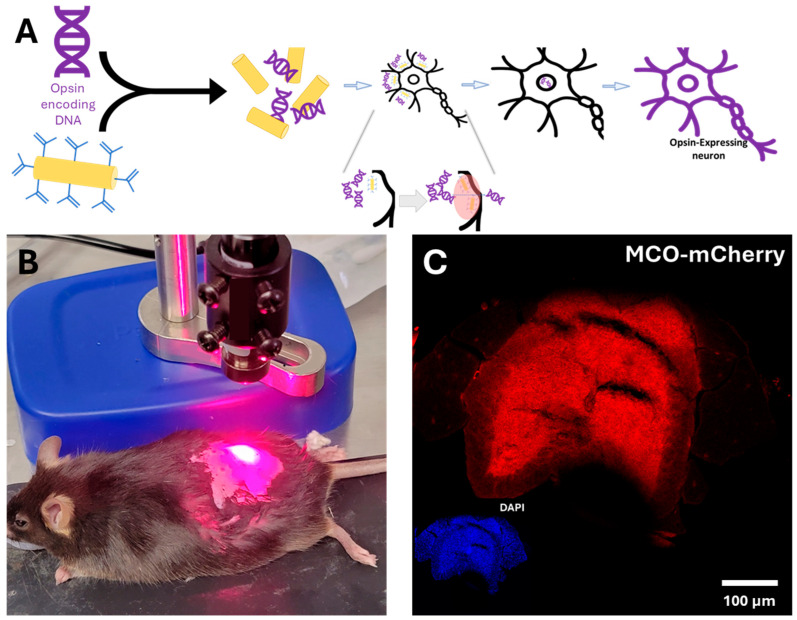
Nano-enhanced Delivery of MCO in the Spine. (**A**) Basic process of Nano-enhanced Delivery. (**B**) Laser irradiation of the lumbar spine to initiate uptake of GAD67-MCO2-mCherry plasmid. (**C**) Inherent mCherry (red) signal reporting MCO expression in the spinal cord of a transfected mouse (Inset: DAPI–blue–image of the transfected spinal cord showing expression is local inside the spinal cord).

Nano-enhanced optical delivery [21] was used to facilitate uptake of the opsin-encoding plasmid into spinal neurons. In this approach, plasmid DNA encoding the GAD67-MCO2-mCherry construct was combined with functionalized gold nanorods (fGNRs, C12 10-650-TC-PBS-50-1-EP Nanopartz, Loveland, CO, USA), which function as photothermal transducers. This methodology enables rapid turnaround and avoids potential immunological responses, which could result in complicating later therapeutic improvements or requiring re-dosing. The overall NOD strategy is illustrated schematically in Figure 1A. Following intrathecal injection, animals were allowed to recover for at least 30 min to permit distribution of the vector throughout the spinal column. After this incubation period, the lumbar region was irradiated with a far-red (650 nm, 650MDLC-100-1342-F-AC Lilly Electronics, Wuhan, China) laser beam (power: 130 mW) with a spot size of 1.5 mm, corresponding to an intensity of 74 mW/mm^2^. The laser irradiation setup used to activate the nanorods and initiate plasmid uptake in spinal neurons is shown in Figure 1B. Activation of the gold nanorods generates localized photothermal effects that transiently increase membrane permeability, thereby promoting cellular internalization of the plasmid DNA.

### 2.5. Pain Scoring (Formalin Test)

Mice received a 20 µL injection of 1% formalin and were subsequently placed in an observation chamber. Pain-related behaviors, specifically paw lifting and paw licking, were monitored for 1 min at 5 min intervals. During each observation period, the cumulative duration (in seconds) of these behaviors was recorded. Observations continued for a total period of 45 min. Pain scores were calculated using the following formula:

$$PainScore=\sum_{i=1}^{n}\frac{b\cdot t}{60}$$

where:

*b* = behavior category weight (lifting = 1 and licking = 2)

*t* = time spent in that behavior (seconds)

The experiment was repeated in wild-type mice both with and without optical stimulation (average power ≈ 167 µW) to evaluate whether stimulation produced differences in behavioral responses. Repeat experiments were kept to a minimum separated from each other to prevent confounding issues such as scar tissue formation or insensitivity due to cumulative damage. Results were analyzed using GraphPad Prism 8.0.2 software utilizing the *t*-test for direct comparison of pain scores between treatment group and baseline responses.

## 3. Results

### 3.1. Nano-Enhanced Delivery of MCO Effectively Transfected the Spinal Cord

Laser-assisted irradiation of the lumbar spinal region was utilized to promote the efficient uptake of the GAD67-MCO2-mCherry plasmid into spinal neuronal tissues. The success of this transfection protocol was rigorously confirmed through the detection of mCherry fluorescence localized within the murine spinal cord (see Figure 1C). To objectively exclude the possibility of autofluorescence confounding the results, DAPI staining was simultaneously performed as a nuclear marker. DAPI, a blue-fluorescent dye, exhibits high affinity for DNA and labels cellular nuclei, while mCherry, a red fluorescent protein, precisely identifies loci of recombinant plasmid expression. DAPI and mCherry fluorescence in the same area were interpreted as the location of MCO expression in the cell membranes of transfected cells, which could be identified via the DAPI signal.

### 3.2. 5 Hz Spinal Optogenetic Therapy Reduces Inflammatory Pain Scores

Optogenetic stimulation at defined frequencies can significantly modulate neural circuits implicated in pain perception. Our prior studies indicate that 5 Hz stimulation effectively attenuates pain-related behaviors within the Anterior Cingulate Cortex [25], a region associated with the affective dimension of pain [26]. The present findings demonstrate that similar effects are observed in the spinal cord. Both in the spinal cord and the brain, inhibitory interneurons are essential for suppressing pain signals by releasing neurotransmitters that inhibit the activity of neurons responsible for transmitting pain [27,28].

**Figure 2:**
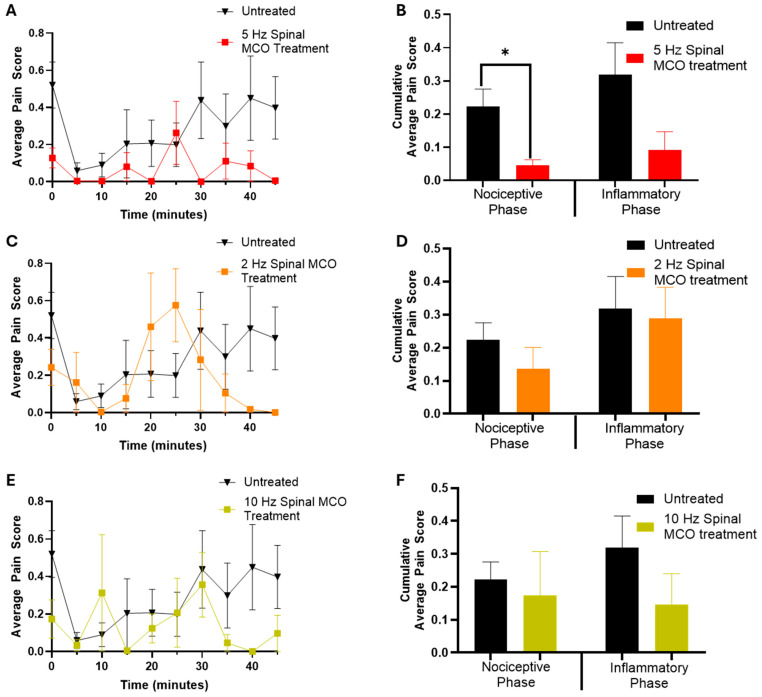
Pain modulation using optogenetic activation of spinal cord is frequency dependent. The 5 Hz optogenetic stimulation (5 ms pulses at 630 nm) of the spinal cord results in reduced pain responses: (**A**) Longitudinal formalin induced pain scores; and (**B**) The average of the cumulative pain scores (measured in 5 min intervals) in the early (0–11 min) non-inflammatory and late (20–41 min) inflammatory phases of pain. The 2 Hz (5 ms pulses at 630 nm) stimulation of the spinal cord does not result in reduced pain responses: (**C**) Longitudinal formalin induced pain scores.; and (**D**) The average of the cumulative pain scores (measured in 5 min intervals) in the early (0–11 min) non-inflammatory and late (20–41 min) inflammatory phases of pain. The 10 Hz (5 ms pulses at 630 nm) stimulation of the spinal cord does not result in reduced pain responses: (**E**) Longitudinal formalin-induced pain scores; and (**F**) The average of the cumulative pain scores (measured in 5 min intervals) in the early (0–11 min) non-inflammatory and late (20–41 min) inflammatory phases of pain. Avg. ± SEM. N = 7 for the untreated and 5 for the spinal treatment groups, respectively. * *p* < 0.05.

The 5 Hz stimulation in the spinal cord (Figure 2A,B) showed significant reductions in quantifiable pain behaviors in the formalin test. The treatment had its most significant effect during the nociceptive phase, corresponding to the early (0–11 min) period (*p* = 0.0299). During this phase, optogenetic stimulation notably decreased pain responses compared to the untreated group, demonstrating the potential efficacy of this approach in reducing acute pain signals. The average pain score was still reduced relative to the untreated results during the phase dominated by inflammatory pain, though possibly due to the variability of inflammatory pain responses, this was not a statistically significant reduction (*p* = 0.0987).

### 3.3. Efficacy of Spinal Optogenetic Therapy Depends on the Stimulation Schedule

The effectiveness of optogenetic stimulation was found to vary according to the frequency of stimulation pulses. In comparison to the previously described 5 Hz protocol, formalin assay results in transfected mice exposed to either 2 Hz or 10 Hz showed a diminished impact on pain inhibition (Figure 2C–F). Analysis of cumulative averages during the nociceptive and inflammatory phases between untreated and treated groups revealed no statistically significant differences in the nociceptive or inflammatory phases. These findings indicate that the efficacy of optogenetic stimulation is closely correlated with pulse delivery frequency.

### 3.4. Sub-Optimal Transfection Lowered the Efficacy

**Figure 3:**
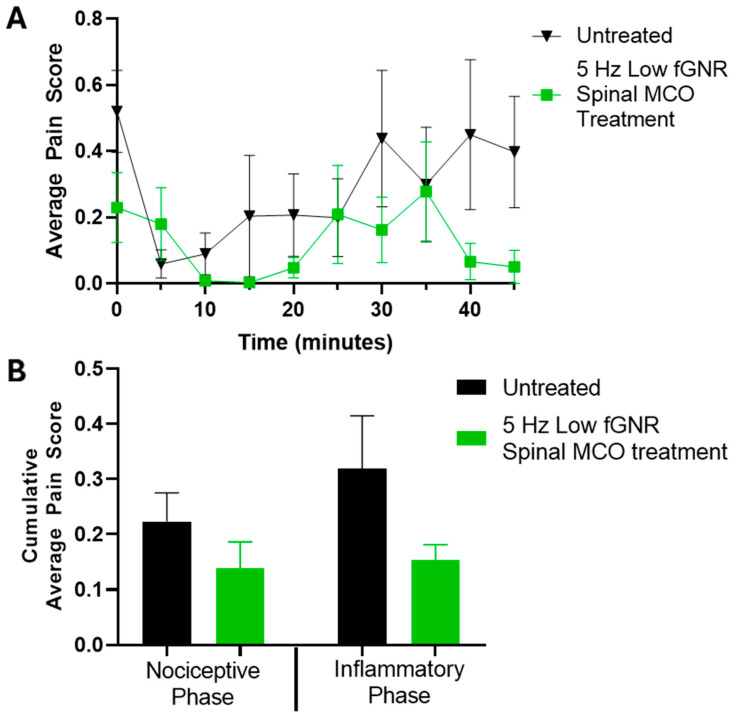
Low efficiency transfection of the spinal cord significantly reduces the effect of 5 Hz optogenetic stimulation on pain responses. After transfection with an fGNR mixture at half the normal concentration (0.2 µg/µL instead of the normal 0.4 µg/µL), the same formalin challenge was performed. (**A**) Longitudinal formalin-induced pain scores. (**B**) The average of the cumulative pain scores (measured in 5 min intervals) in the early (0–11 min) non-inflammatory and late (20–41 min) inflammatory phases of pain. Avg. ± SEM. N = 7 for the untreated and 4 for the spinal treatment groups, respectively.

To evaluate the impact of transfection efficiency on optogenetic modulation of pain, the concentration of the fGNR–plasmid mixture used for nano-enhanced delivery was reduced to half of the standard level (0.2 µg/µL instead of 0.4 µg/µL) prior to intrathecal injection. Behavioral responses were subsequently assessed using the formalin pain assay following optogenetic stimulation at the previously identified optimal frequency of 5 Hz. Longitudinal formalin-induced pain scores recorded during the 45 min observation period are shown in Figure 3A. Mice receiving the reduced concentration of GNRs exhibited higher pain scores with optogenetic activation as compared to animals having the double concentration of GNRs.

Cumulative pain scores calculated for the early nociceptive phase (0–11 min) and the later inflammatory phase (20–41 min) are presented in Figure 3B. Animals with reduced concentration of GNRs demonstrated a smaller reduction in pain-related behaviors following optogenetic stimulation compared with those transfected with the standard (0.4 µg/µL) concentration of GNRs.

## 4. Discussion

The spatial distribution of DAPI and mCherry fluorescence signals demonstrated robust expression in the targeted region of the spinal cord. This demonstrated that the nano-enhanced optical delivery (NOD) methodology is an efficient and reliable method to deliver genetic material in the spinal cord. When compared to viral delivery methods, NOD offers more rapid, effective expression of opsins, trading systemic reach for high spatial precision, an attractive option for sensitive, potent neuromodulation targets like the spinal cord or deep nuclei [29]. However, as a novel methodology, there is room for more development that will enhance our understanding and the success and ease of delivery. By providing a rapid turnaround from dose to expression and avoiding the generation of neutralizing antibodies to a viral vector, this technique keeps many possible options open for human translation and improves the speed at which effective targeted therapy can begin.

The diminished optogenetically modulated behavioral response observed under reduced transfection conditions also indicates that the therapeutic impact of optogenetic stimulation is intricately linked to the efficiency of gene delivery. When the concentration of the gold nanorods (that enhances the optical delivery) was lowered, the resulting pain modulation effect was reduced. This likely reflects reduced opsin expression in spinal neurons, which would decrease the number of cells capable of responding to optical stimulation. As a result, the overall neural response to light stimulation may be attenuated, leading to smaller reductions in pain-related behaviors. These observations highlight the importance of optimizing transfection efficiency, alongside stimulation parameters, when developing optogenetic neuromodulation strategies for therapeutic applications.

The observed frequency-dependent effect with optogenetic stimulation underscores the fundamental role that specific patterns of neuronal activation play in regulating nociceptive processing across multiple regions of the central nervous system. Only the 5 Hz protocol yielded considerable analgesic effects, suggesting an optimal stimulation window required for effective pain modulation via optogenetics. Lower frequencies, such as 2 Hz, may not activate the relevant neural circuits sufficiently, whereas higher frequencies like 10 Hz could induce desensitization or confounding asynchronous action potentials, thereby reducing efficacy. The higher efficacy of 5 Hz optogenetic stimulation suggests potential for frequency-dependent intervention strategies that could be utilized in therapeutic settings to manage chronic or acute pain conditions. Importantly, the function of inhibitory interneurons extends beyond simple signal attenuation; they orchestrate complex neural network dynamics that ensure precise control over sensory input. This frequency-dependent pattern underscores the importance of carefully optimizing stimulation parameters for experimental and potential clinical use in controlling nociceptive responses.

The observed reduction in pain-related behavior following optogenetic stimulation supports the efficacy of optogenetic neuromodulation via nano-enhanced optical delivery of opsin-encoding genes to central neurons. The success of the delivery depends on the confluence of light delivery and the proper local concentration of plasmids and GNRs, as suboptimal concentrations do not result in effective expression of opsins in the spinal cord. The success of optogenetic stimulation requires a proper attunement of the frequency and intensity of optogenetic stimulation light bursts in the spinal cord. It is interesting to note that the effectiveness of optogenetic stimulation with 5 Hz stimulation was primarily seen in the nociceptive phase rather than the inflammatory phase. This may be related to the limited number of experimental animals, rapid experimental turnaround from gene delivery (2 days after NOD) before MCO expression was able to reach a therapeutic threshold, and the variability of response intensity during the inflammatory phase of the formalin test.

Compared with conventional neuromodulation techniques, optogenetic approaches offer several potential advantages, including improved cellular specificity and reduced reliance on invasive electrode implantation. Because optogenetic approaches allow selective activation of defined neuronal populations, they are well-suited for addressing the heterogeneous mechanisms that contribute to chronic pain. Our studies using optogenetics-based spinal stimulation in a murine model demonstrated significant reductions in reflexive pain behaviors, supporting the potential therapeutic utility of this modality. The implementation of MCO-based optogenetic modulation further enhances these benefits by enabling cell-type-specific stimulation with low optical power requirements. The high sensitivity of the MCO system allows the use of lower light intensities, which reduces the risk of tissue heating or phototoxic effects while maintaining effective neural activation.

## 5. Conclusions

In summary, our findings demonstrate that nano-enhanced optical delivery of MCO enables robust expression of opsin for effective neuromodulation of spinal pain circuits in murine models. The 5 Hz stimulation significantly reduced pain-related behaviors, particularly during the acute nociceptive phase. Responses were not significantly affected if the stimulation was below the typical frequency (2 Hz) or well above it (10 Hz). This lack of efficacy continued when the NOD parameters were affected by a lack of photothermal transducers. Optogenetic stimulation produced quantifiable reductions in pain-related behaviors, with therapeutic outcomes dependent on both gene delivery efficiency and stimulation parameters. These results support the potential of spinal optogenetic neuromodulation as a promising platform for future development of next-generation, cell-specific pain therapies.

To move this technology closer to clinical application, the next stage of development should concentrate on scaling and validating these findings in larger, statistically robust cohorts to ensure the reproducibility and longevity of the observed effects. Furthermore, studies in larger animal models are crucial, as our current approach is limited by the relatively permissive nature of murine tissues for gene delivery. For clinical translation, the delivery strategy will need to closely resemble what is feasible in human patients. Therefore, refinement is required to better match the anatomical and physiological requirements of clinical scenarios. Using models with spinal anatomy and tissue volumes more representative of humans will be vital for optimizing delivery methods, determining light penetration and dosing parameters, and evaluating device or vector performance under conditions relevant to future clinical use.

Equally important is the implementation of a comprehensive preclinical safety program, including expanded assessments of biodistribution, immunogenicity, long term expression stability (building on that shown in NHP by Sharif et al., 2025 [30]), and potential off target effects. Rigorous toxicology and efficacy testing, aligned with regulatory expectations for gene-based neuromodulation therapies, will be required to establish a robust foundation for future first in human studies. Together, these steps will accelerate the progression of nano enhanced spinal optogenetics from a promising experimental approach to a workable, clinically translatable strategy for targeted pain intervention.

## Data Availability

The raw data supporting the conclusions of this article will be made available by the authors on request.
